# The Human Fovea Is Relatively Horizontally Elongated in Infantile Nystagmus

**DOI:** 10.1167/iovs.66.14.56

**Published:** 2025-11-21

**Authors:** Nikita Thomas, Jennifer H. Acton, Jonathan T. Erichsen, James Fergusson, Nick White, Matt J. Dunn

**Affiliations:** 1 School of Optometry and Vision Sciences, Cardiff University, Cardiff, United Kingdom; 2 Vision and Hearing Sciences Research Centre, Anglia Ruskin University, Cambridge, United Kingdom

**Keywords:** fovea, idiopathic, optical coherence tomography

## Abstract

**Purpose:**

Infantile nystagmus (IN) is characterized by primarily horizontal, repetitive eye movements. IN develops in the first few months of life, in tandem with the postnatal development of the fovea. This study tested the hypothesis that the foveal pit is horizontally elongated in adults with IN, corresponding to the streak of the retina over which the image constantly oscillates.

**Methods:**

In 12 adults with IN (without associated conditions known to affect the fovea) and 12 healthy controls (age, sex, and ethnicity-matched), horizontal and vertically-orientated foveal images were acquired with a long-wavelength (λ_c_ 1040 nm) optical coherence tomography system. Horizontal and vertical foveal pit diameters were measured and the ratio between them (foveal shape factor).

**Results:**

Foveal shape factor (vertical/horizontal pit diameter ratio) was significantly lower (more horizontal) in participants with IN compared to controls (0.89 vs. 0.96, *P* = 0.006, BF_10_ = 4.14).

**Conclusions:**

IN is associated with significant relative horizontal elongation of the anatomical fovea. These results indicate that early-onset nystagmus and foveal structure could have a direct impact on each other during development. Future work should investigate whether these differences are present at the photoreceptor level.

Infantile nystagmus (IN) is characterized by a predominantly horizontal, conjugate involuntary movement of the eyes.^1^^–^^3^ IN appears within the first few months after birth,^4^ persists for the rest of life, and visual acuity (VA) is often reduced.^2^^,^^3^ IN is frequently associated with visual system disorders such as albinism and achromatopsia.^3^ These conditions are generally accompanied by a poorly formed or entirely absent foveal pit, known as foveal hypoplasia.^5^ However, IN can also occur without any detectable comorbid visual pathology, which is known as idiopathic IN. Although structural defects of the retina have been shown in various conditions associated with IN (e.g., albinism, achromatopsia, PAX6 mutations),^5^ the direct relationship between IN eye movements and retinal structure has not previously been investigated.

IN eye movements gradually appear at an average age of just over eight weeks from birth,^4^ while the fovea is still undergoing excavation and maturation. Morphologically, the site of the future fovea can be identified as early as 22 weeks gestational age by a cone-only photoreceptor layer and a thick layer of ganglion cells.^6^^,^^7^ By 28–29 weeks gestational age, the foveal pit becomes more clearly defined with prominent thinning of the inner nuclear layer.^6^ After birth, the pit becomes shallower and wider, with full maturity achieved by 15–45 months of age.^7^

We predicted that foveal shape would be “stretched” in the horizontal axis in people with IN, based on considerations specific to this group:

1. Nystagmus is typically horizontal^3^
2. Visual information is continuously available throughout the nystagmus waveform^8^

Taken together, this implies that the locus of visual attention oscillates across a broader area of the retina in IN than in typical observers, which might therefore result in a horizontally “stretched” foveal shape.

In a normal population, foveal pit diameter is known to be slightly larger horizontally than vertically.^9^ Although studies have evaluated retinal layer thicknesses and foveal parameters (e.g., foveal pit depth and foveal pit height) in IN and associated conditions,^10^^–^^12^ to date, horizontal versus vertical foveal shape has never been investigated in an IN cohort.

This study represents an exploratory investigation into horizontal and vertical foveal parameters in adults with IN, using structural optical coherence tomography (SD-OCT). The study was designed to estimate effect sizes and identify patterns that could inform future confirmatory work. A secondary aim was to investigate the relationship between foveal shape and eye movement characteristics. As described above, we hypothesized that foveal pit diameter would be horizontally elongated in individuals with IN, and that any such elongation might be related to nystagmus waveform shape or amplitude.

## Methods

### Participants

Twelve participants with idiopathic IN were recruited from the Cardiff University Research Unit for Nystagmus cohort (total following exclusions; see paragraph below), and 12 healthy control participants were recruited from the School of Optometry and Vision Sciences at Cardiff University, each of which was matched by age (to within five years), sex, and ethnicity. Such matching was important, because the overall thickness of the macula/fovea varies with age, sex, and ethnicity.^13^^–^^16^ The investigation was carried out in accordance with the Declaration of Helsinki; informed consent was obtained from the participants after explanation of the nature and possible consequences of the study. The Cardiff University School of Optometry and Vision Sciences Research Ethics Audit Committee granted approval for this study (reference number 1438).

Participants with IN were recruited based on a prior clinical diagnosis of idiopathic IN established by an ophthalmologist after full clinical evaluation, including normal electroretinography and visual evoked potential testing. These clinical diagnoses were recorded in our institutional database and formed the basis for participant eligibility. Given the growing recognition of genetic heterogeneity within idiopathic IN, and these clinical diagnoses often predated recent advances in the identification of relevant genetic mutations, all participants were offered genetic testing to support accurate classification. Five individuals were excluded from the final analysis after the identification of pathogenic mutations in the following genes known to affect foveal development or ocular morphology: *FRMD7*, *PAX6*, *AP3B1*, *SLC45A2*, and *BLOC1S3*. Three additional individuals were excluded because of the presence of associated conditions, specifically congenital cataracts and optic nerve coloboma.

Although genetic testing was offered, it was not mandatory for study participation, and several individuals either declined testing or did not complete follow-up, resulting in incomplete genetic data for some participants. Although all included participants had been *clinically* diagnosed with idiopathic IN, the possibility remains that some may harbor undetected pathogenic variants and a degree of foveal hypoplasia. However, it is important to note that the clinical definition of idiopathic IN allows for the presence of mild foveal hypoplasia in some cases.^5^ Accordingly, complete exclusion of all structural foveal variation within such a cohort is unlikely. Each participant's level of foveal hypoplasia was graded according to the Leicester Grading Scale for Foveal Hypoplasia^10^ and is displayed in Table 1. VA was measured at a distance of 4 m using an Early Treatment Diabetic Retinopathy Study (ETDRS) chart, with participants given as long as they wish to respond, and to adopt their null zone, if present. The mean VA of participants with IIN was 0.46 logMAR; within half a standard deviation of the mean reported in a cohort of 133 patients with IIN.^3^

**Table 1.** Clinical Data for Participants With IN

							Primary Position Waveform			
Participant	Age/Sex	Refraction	Clinical VA (logMAR)	Eye Tested	Foveal Hypoplasia?^39^	Foveal Shape Factor	Type	Axis	Amplitude	Frequency (Hz)
P016	34/M	R: 1.25/−3.00 × 161	R: 0.50	R	Grade 1	0.938	BDJR	Horizontal	0.7°	1.8
		L: −2.50/−3.00 × 32	L: 0.52							
P003	67/M	R: 1.25/−0.75 × 20	R: 0.48	R	No	0.829	RPC	Horizontal	4.9°	2.0
		L: −0.50/−0.25 × 140	L: 0.48							
P013	65/M	R: −2.00/−1.50 × 148	R: 0.80	R	Grade 1	0.950	JR_EF_	Horizontal	1.2°	4.8
		L: −3.25/−0.75 × 155	L: 0.80							
P037	22/F	R: plano/−0.25 × 15	R: 0.10	R	No	0.947	JR	Horizontal	0.3°	1.9
		L: 0.75/−1.00 × 55	L: 0.16							
P025	46/F	R: −2.00/−0.25 × 10	R: 0.48	R	No	0.968	BDJR	Horizontal	1.3°	1.7
		L: −1.00/−1.25 × 135	L: 0.60							
P028	60/F	R: −4.00/−2.00 × 32	R: 0.52	L	No	0.938	J_EF_ (PAN)	Horizontal	3.3°	1.6
		L: −2.25/−5.25 × 20	L: 0.50							
P004	47/M	R: 0.50/−0.25 × 180	R: 0.10	L	No	0.794	PP_FS_	Horizontal	0.8°	1.3
		L: 0.25/−0.25 × 180	L: 0.08							
P011	45/M	R: −1.25/−2.50 × 5	R: 0.48	R	No	0.643	PP_FS_	Horizontal	4.8°	0.7
		L: −6.75/−1.25 × 175	L: 1.00							
P048	22/F	R: 3.00/−3.50 × 10	R: 0.30	L	Grade 1	0.867	JL	Horizontal	1.3°	1.7
		L: 2.25/−2.50 × 5	L: 0.24							
P010	60/F	R: −1.00/−2.50 × 175	R: 0.72	R	No	0.931	J_EF_ (PAN)	Horizontal	1.3°	0.9
		L: −1.25/−2.00 × 160	L: 0.74							
P052	34/M	R: −1.00/−0.50 × 170	R: 0.44	R	Grade 1	0.980	DJ (PAN)	Horizontal	6.7°	1.8
		L: −1.25/−0.75 × 150	L: 0.44							
P051	36/F	R: 3.50/−1.50 × 25	R: 0.28	R	No	0.920	JR_EF_	Horizontal	2.5°	1.2
		L: 2.25/−1.00 × 20	L: 0.30							

BDJ(R/L), bidirectional jerk; DJ, dual jerk; J(R/L)_EF_, jerk with extended foveation; J(R/L), pure jerk; L, left; PAN, periodic alternating nystagmus; PJ(R/L), pseudo jerk; PP(_FS_), pseudo pendular (with foveating saccades); R, right; RPC, pseudo cycloid.

Note that each participant with nystagmus was age, sex and ethnicity-matched to a control participant.

The inclusion criteria for matched control participants were: corrected VA of 0.00 logMAR or better, mean refractive error ≤ ±6.00 D sphere in any meridian in the test eye, intraocular pressure ≤ 24 mm Hg as measured with a non-contact tonometer (Pulsair Desktop Tonometer; Keeler Ophthalmic Instruments, Windsor, UK), and lenticular opacities no greater than grade two in any category of the Lens Opacity Classification System (LOCS III)^17^. No participants had prior cataract surgery. Normal optic nerve head and fundus appearance were confirmed in matched controls by an optometrist using fundoscopy and clinical OCT images. Iris transillumination (a hallmark of ocular/oculocutaneous albinism) was ruled out in all participants, and the presence of IN was confirmed via identification of foveations and an accelerating waveform in eye movement recordings (see “Eye tracking”). The presence of a null zone/convergence null zone was also established in all participants, as well as assessment of any latent component. Eye movements were examined for a reversal of beat direction with alternate occlusion to confirm the absence of fusion maldevelopment nystagmus syndrome in the participants with IN. Participants (IN and matched controls) with a history of glaucoma, family history of glaucoma, diabetic retinopathy, ocular hypertension, ocular trauma, and those taking medications known to affect visual function were excluded.

### OCT Imaging

The eye with the better VA was chosen as the test eye or, if both eyes had equal VA, the right eye was imaged by default. One drop of Tropicamide 1% was instilled into the eye if deemed clinically necessary (e.g., due to pupil miosis or early lens opacities) to achieve acceptable SD-OCT image quality. Axial eye length (cornea to retinal pigment epithelium) was measured using optical biometry (IOL Master, Carl Zeiss Meditec, Dublin, CA) in order to scale the processed SD-OCT images correctly. An axial eye length signal-to-noise ratio above 2.0 was considered sufficient (IOL Master Manual V.5 2007, Carl Zeiss Meditec, Dublin, CA). SD-OCT images were qualitatively inspected with manual re-focusing and re-positioning to ensure good image quality. This involved real-time joystick-controlled adjustment of focus and lateral beam position while viewing the live OCT and fundus image display, enabling optimal alignment on the fovea before capture. In participants with nystagmus, multiple acquisition attempts were made, with dynamic beam positioning and re-focusing between acquisitions to obtain the best possible images despite continuous eye movement.

SD-OCT images were obtained from all participants using a non-commercial long-wavelength (λ_c_ 1040 nm) SD-OCT instrument, established by Považay et al.^18^ and utilized previously.^19^^–^^21^ The system operated at an imaging rate of 47,000 A-lines/s, with an axial resolution of ∼5 µm in tissue (refractive index = 1.4) and a theoretical lateral resolution of ∼6 µm. All images were obtained by a single trained operator (author NT).

Horizontally-orientated scans spanned 20 × 0.625° and vertically-orientated scans spanned 0.625 × 20° (horizontal and vertical scans were taken separately), with 1024 pixels in each A-line, 1024 A-lines in each B-scan, and 32 B-scans in each volume. This was completed eight times across the same foveal area, resulting in eight volumetric images of 32 × 1024 × 1024 pixels. This scan protocol was specifically designed for foveal imaging in the presence of IN. The different orientations ensured that the A-lines (the fastest part of the scan) were aligned and took precedence in horizontal (0°) and vertical (90°) scans. The small scan angle (0.625°) was selected to allow for some patient motion and set up error, which facilitated identification of the image acquired directly through the foveal center. Two repeats of the scan protocol were acquired in the test eye; the capture time for the entire scan protocol was 5.58 seconds. Discussion of the potential impact of IN eye movements on the acquired images is provided later in this article.

### Eye Tracking

To measure waveform parameters in participants with IN, binocular horizontal and vertical gaze position was recorded in screen-based (pixel) co-ordinates for five minutes at 1000 Hz by an EyeLink 1000 Plus infrared video eye tracker (SR Research, Ottawa, ON, Canada; firmware version 5.12) using a Tower Mount. The eye tracker was calibrated, and nystagmus waveforms segmented for analysis, using the method described by Dunn et al.^22^ The head was stabilized by a chin and forehead rest. A 2° red fixation cross^23^ was presented in the primary position (straight ahead) at 33 cm on cathode-ray tube display (Sony Trinitron CPD-G520, 100 Hz, 21”, with a luminance range of 0.049–84.64 cdm^−2^). Room lights were extinguished prior to stimulus presentation.

### OCT Image Analysis

SD-OCT foveal images were processed and exported into ImageJ (Rasband; National Institute of Health, Bethesda, MD, USA)^24^ to undergo contrast enhancement with a 3 × 3 median filter to reduce speckle noise. Images exhibiting acquisition tilt were also digitally rotated in post-processing. Rotation was applied around the z-axis such that the retinal pigment epithelium was aligned horizontally with the x-axis of the image coordinate system (“TransformJ” package). Images were then imported into OCT Explorer 3.8 (Retinal Image Analysis Lab, Iowa Institute for Biomedical Imaging, Iowa City, IA, USA) and segmented using the Iowa Reference Algorithms.^25^^,^^26^

### Image Scaling and Extraction of Foveal Parameters

Because of the continuous IN eye movements, it was necessary to include many B-scans (256 in the present study; 8 × 32) to ensure that at least one scan imaged the foveal center. Because this study was primarily concerned with extracting horizontal and vertical parameters at the center of the fovea, parameters only needed to be extracted from the B-scan containing the foveal center or lowest retinal thickness at the center of the foveal pit (RtFP; also known as “foveal height”).

### Identification of Foveal Center

In individuals with stable fixation, most foveal OCT volume scans can image the foveal center with high accuracy. However, even small errors in central fixation can reduce the reliability of manually detecting the foveal center.^27^ This is exacerbated in individuals with IN, in which the location accuracy of B-scans is further reduced because of unstable fixation. Given the lack of reliability when using qualitative observation to determine the foveal center in individuals with IN, a quantitative method was developed. The image containing the foveal center always has the lowest retinal thickness at the center of the foveal pit (RtFP; distance between the foveal pit at the inner limiting membrane [ILM] and the photoreceptor layer).^28^ To eliminate subjective bias in image selection, a MATLAB program selected the foveal B-scan from each set with the lowest RtFP. This program isolates 25-pixel windows across the ILM, with each subsequent window shifted by one pixel (i.e., first window: pixel 1–pixel 25; second window: pixel 2–pixel 26, etc.). A line of best fit (ordinary least squares) is determined for each 25-pixel window, and a line normal to the line of best fit is drawn to the photoreceptor layer (inner segment/outer segmented layer). This iterative process continues along the entire length of the segmented data. The normal line with the shortest length is then identified as the RtFP and compared with the 255 other images to determine the image with the lowest RtFP overall. This was performed for both horizontal and vertical scans separately. Although this method was particularly important for SD-OCT images acquired in the presence of IN, for consistency the method was also used for images acquired from control participants.

### Astigmatic Correction

Astigmatic axis and power can distort the OCT retinal image and affect its size.^29^ This may be particularly problematic when investigating measured parameters in a specific dimension, as is the case in the present study. Following lateral scaling to convert the original pixel values in SD-OCT images to micrometers (as well as correcting for transverse magnification), astigmatism correction factors (*q_major_* and *q_minor_*) were calculated using the equations described by Langenbucher et al.^30^ These correction factors were then used in the equation *t* = *pqs*^31^^,^^32^ to determine the true anatomical dimension of a retinal feature from the measured OCT dimension, where:

*t* = true dimension of a retinal feature
*p* = magnification correction factor related to the imaging system
*q* = magnification correction factor related to the eye
*s* = the measured OCT dimensions of a retinal feature

For participants with with-the-rule astigmatism, *q_major_* was applied to measurements from horizontally-orientated scans, and *q_minor_* was applied to measurements from vertically-orientated scans (vice versa for against-the-rule astigmatism). Because with-the-rule astigmatism is particularly common in individuals with IN,^33^ this additional correction reduced the likelihood of optical artefacts affecting measurements obtained from the SD-OCT images.

### Foveal Measurement

**Figure 1.**
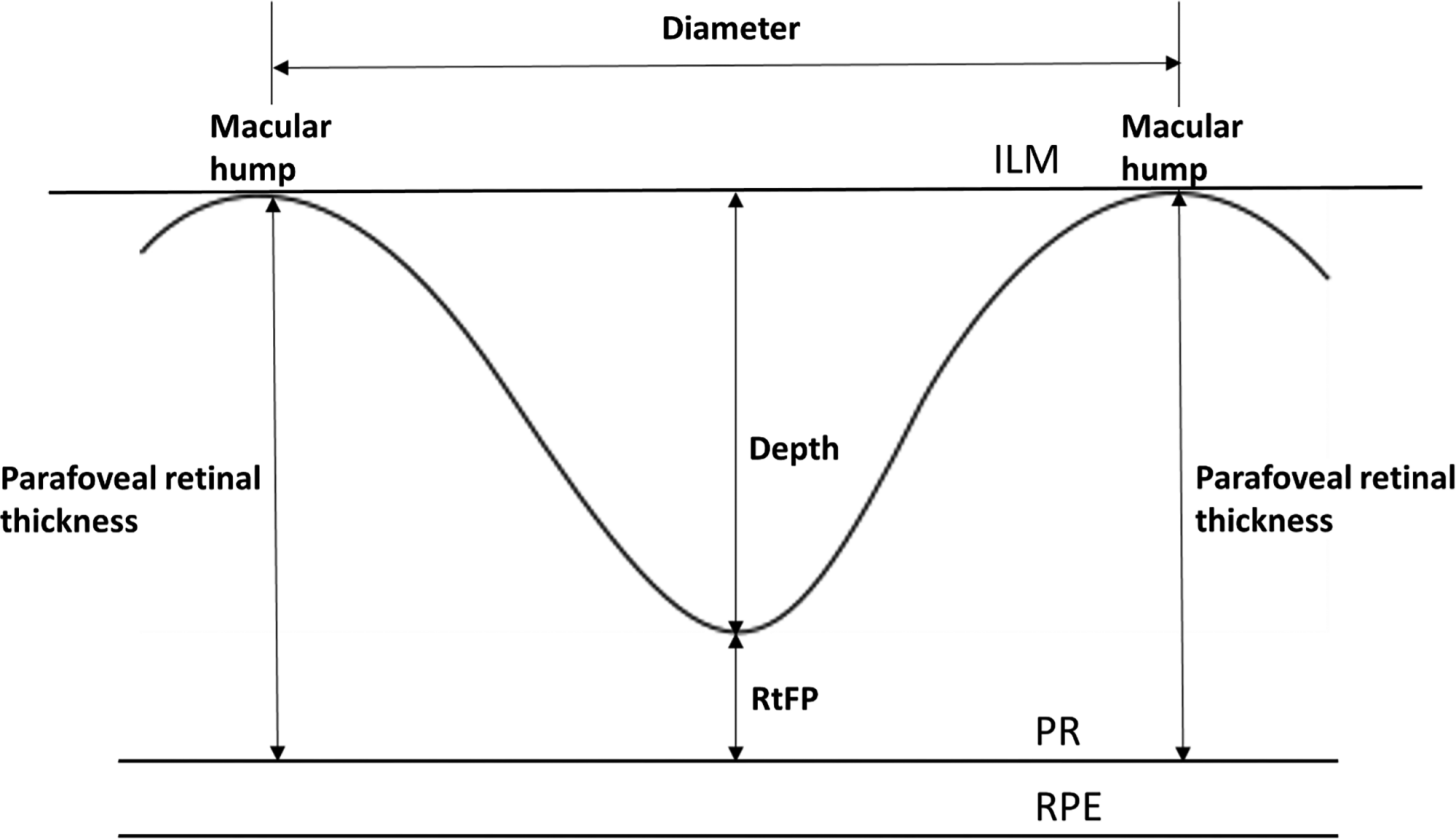
A cross-sectional foveal diagram portraying parameters extracted from images with the lowest retinal thickness at the center of the foveal pit (deepest foveal pit). PR, photoreceptor layer; RPE, retinal pigment epithelium.

The horizontal and vertical images identified with the lowest RtFP were then processed to extract foveal pit diameter and the parafoveal retinal thicknesses highlighted in Figure 1. Macular hump positions were determined automatically by a MATLAB program (version 2018a; The MathWorks, Inc., Natick, MA, USA), which analyzed the segmented ILM data pixel-by-pixel, starting from the center of the foveal pit. The program identified the highest pixel value on either side of the foveal pit within the pericentral 3000 µm ring of the ETDRS macular grid (i.e., the inner ring subfield).^34^ Images were scaled for axial eye length to remove the effect of transverse magnification (a feature present in all OCT images) and to convert measurements from pixels to micrometers.^31^^,^^35^

Axial scaling is only affected by the refractive index of the medium of interest and, consequently, was the same for all eyes.^36^ The axial scaling factor for the long-wavelength SD-OCT instrument used in the present study was 1.93 µm per pixel in tissue.

### Statistical Analysis

Statistical analyses were performed using JASP.^37^ Any *P* value less than 0.05 was considered significant and, when displayed, is shown alongside effect sizes (Cohen's *d* for *t*-tests and rank-biserial correlation for Mann-Whitney U tests) and their associated confidence intervals. Throughout the analysis of the present study, Bayes factors (BF_10_; prior scaling parameter = 0.707) are also given alongside frequentist statistics for reference (for an introduction to Bayes factors, see Kass and Raftery^38^).

To test the hypothesis that foveal pit diameter is horizontally elongated in adults with IN compared to controls, shape factor was calculated from the foveal pit diameter measurements, defined as the vertical/horizontal foveal pit diameter ratio, where a shape factor < 1.0 indicates horizontal elongation, and a shape factor > 1.0 indicates vertical elongation. A two-tailed independent samples *t*-test was used to compare foveal shape factor between participants with IN and controls.

Accurate SD-OCT image capture of the foveal center was identified through confirmation that the measurement value of RtFP from the selected horizontal B-scan was similar to that from the vertical scan. A two-tailed paired samples *t*-test was used to compare horizontal and vertical RtFP within each participant group.

**Figure 2.**
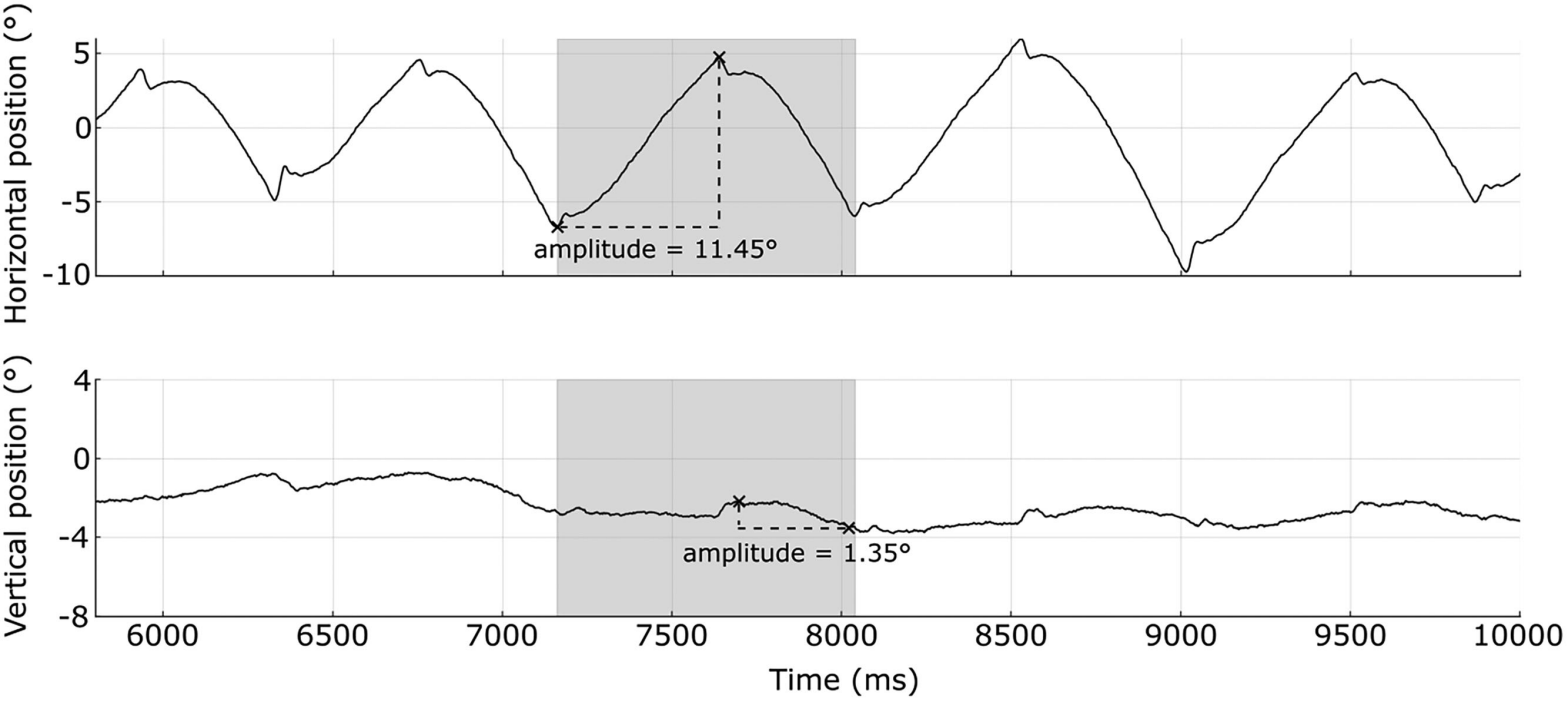
Example horizontal (*top*) and vertical (*bottom*) eye position traces from participant P011 with idiopathic IN. The *shaded region* indicates a single cycle, with *black crosses* displaying the minimum and maximum positions. The waveform shape factor was derived as the median vertical/horizontal position ratio across all cycles.

*Waveform shape factor* was also calculated for each participant with IN. This was determined by first finding the range of horizontal and vertical eye positions produced within each IN waveform cycle, followed by calculation of the vertical/horizontal ratio of these ranges (see Fig. 2 for an example). The median value across all nystagmus cycles was taken as the *waveform shape factor*. Similar to the calculation of foveal shape factor, a waveform shape factor < 1.0 indicates predominantly horizontal eye movements, and a waveform shape factor > 1.0 indicates predominantly vertical eye movements.

To investigate the secondary aim regarding the relationship between foveal shape and characteristics of the IN waveform, multiple regression analyses were performed for horizontal IN amplitude, waveform shape factor, and VA. Horizontal foveal pit diameter and foveal shape factor were used as predicting variables in each regression model. The Shapiro-Wilk test was performed prior to performing *t*-tests; if any variables showed a significantly non-normal distribution (*p* < 0.05), a Mann-Whitney U test was performed instead.

## Results

Clinical details for participants with IN are shown in Table 1. Images from three participants with IN and one matched control required very minor manual adjustments to the overall segmentation (<2 layer segmentation boundaries were manually adjusted in certain parts of the selected image to keep segmentation consistent across the image).

### Horizontal and Vertical Parameter Comparisons

**Figure 3.**
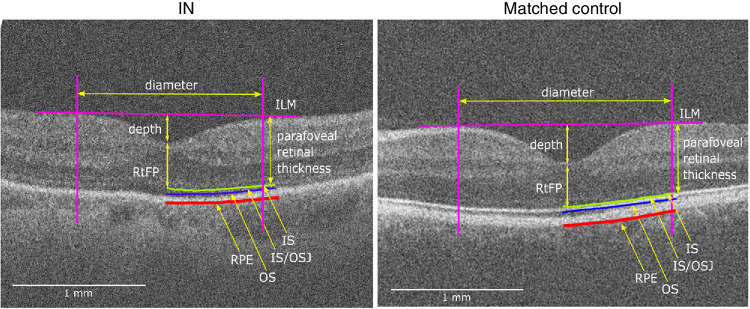
Examples showing the foveal parameters extracted from a participant with IN (age 34; P052) and a matched control (age 30). IS, inner segment (photoreceptors); IS/OSJ, inner segment/outer segment junction (photoreceptors); OS, outer segment (photoreceptors); RPE, retinal pigment epithelium.

Figure 3 illustrates an example of the foveal parameters extracted from a participant with IN and a matched control. A paired samples *t*-test found no significant difference between horizontal and vertical RtFP within participants with IN (166 µm vs. 168 µm; *P* = 0.21, *t* = −0.88, *d* = −0.25 [95% CI, −0.82 to 0.33], BF_10_ = 0.40) and within matched controls (151 µm vs. 151 µm; *P* = 0.49, *t* = −0.72, *d* = −0.21 [95% CI, −0.78 to 0.37], BF_10_ = 0.36).

Table 2 presents horizontal and vertical foveal parameters extracted from participants with IN and controls. All parameters were significantly larger in participants with IN, except for foveal pit depth and foveal pit diameter (both horizontal and vertical). RtFP measurements are similar to those reported previously in both controls and IIN.^40^

**Table 2.** Horizontal and Vertical Foveal Parameters (Mean ± SE; µm) in Participants With IN and Matched Controls

	IN	Matched Controls	*P* Value
RtFP (average of horizontal and vertical)	167 ± 6	151 ± 3	0.027
Foveal pit depth (average of horizontal and vertical)	76 ± 5	113 ± 2	<0.001
Horizontal foveal pit diameter	1821 ± 129	2098 ± 34	0.0501
Vertical foveal pit diameter	1608 ± 104	2022 ± 33	0.001
Nasal parafoveal thickness	244 ± 5	264 ± 3	0.001
Temporal parafoveal thickness	239 ± 4	260 ± 4	0.001
Superior parafoveal thickness	245 ± 5	268 ± 3	<0.001
Inferior parafoveal thickness	243 ± 4	263 ± 3	<0.001

Because there was no significant difference between horizontal and vertical RtFP or foveal pit depth, the values for these are averaged across horizontal and vertical measurements. Independent-samples *t*-test *P* values are shown (Holm-Bonferroni-corrected; note that *superior parafoveal thickness* [only] uses a Mann-Whitney U test because of data non-normality); all are significant.

### Foveal Shape Factor Analysis

To address the main hypothesis, foveal shape factor (vertical/horizontal foveal pit diameter ratio) was calculated for each participant and averaged across all participants in each group. Foveal shape factor was significantly lower (i.e., the foveal pit diameter was more horizontally elongated) in participants with IN compared to controls (Mann-Whitney U test [0.89 vs. 0.96; *P* = 0.006, U = 119, *r* = −0.65 {95% CI, −0.30 to −0.85}, BF_10_ = 4.14]).

### Effect of Nystagmus During OCT

The use of a custom-built OCT device enabled us to rotate the sweep orientation. A-lines were always obtained in the same direction as the image being produced (e.g., when obtaining vertical images of the fovea, the light source was swept vertically). Unlike most commercial OCT systems, this ensured near-instantaneous acquisition in both axes. Nevertheless, nystagmus eye movements have the potential to skew the position of the A-lines within each B-scan, resulting in artefactual elongation or contraction of foveal pit diameter in the horizontal dimension.

To investigate this potential source of error, for each participant with IN, the typical amplitude of horizontal eye movement was determined (from eye movement recordings obtained during the central fixation task) for the duration of a single B-scan acquisition (21.8 ms) and converted to microns of retinal shift. Each IN participant's horizontal foveal pit diameter was adjusted for the average horizontal eye movement during a B-scan in each participant with IN. Foveal shape factor (for the B-scan containing the foveal center) was recalculated following these adjustments. Across all participants with IN, the average variation in foveal shape factor due to horizontal IN eye movements was ±0.006. This variation encompasses both potential artefactual elongation (*with* the direction of IN eye movements) and contraction (*against* the direction of IN eye movements) of the foveal pit diameter. To determine the extent to which the difference in shape factors found in the present study might be explained by an artefactually elongated/contracted B-scan due to the eye movements, another two-tailed independent samples t-test was performed on the same data, with foveal shape factors adjusted to the maximum that could be explained by artefactual image elongation (i.e., a worst-case scenario). Even with this adjustment, the difference in foveal shape factor between groups (0.90 [nystagmus] vs. 0.96 [controls]) remained significant (*P* = 0.012, U = 115, *r* = −0.60 [95% CI, −0.21 to −0.82], BF_10_ = 2.82), indicating that our findings cannot be explained solely by artefactual image elongation.

### Relationship Between Horizontal Foveal Pit Elongation and IN Waveform Parameters

The maximum vertical range of eye positions across all cycles from participants with IN was 2.12°, and the maximum horizontal range was 11.51° (i.e., IN was predominantly horizontal). Multiple regression models predicting horizontal IN amplitude, waveform shape factor, and VA from horizontal foveal pit diameter and foveal shape factor are shown in Table 3. All models were not significant with low adjusted *R*^2^ values, and neither horizontal foveal pit diameter nor foveal shape factor were significant predicting variables for horizontal IN amplitude, waveform shape factor, and VA.

**Table 3.** Multiple Regression Models Predicting Horizontal Nystagmus Amplitude, Waveform Shape Factor, and VA From Horizontal Foveal Pit Diameter and Foveal Shape Factor

Outcome Variable	Adjusted *R*^2^	F_2,12_	*P*	Predicting Variables	Coefficient	*t*	*P*
Horizontal nystagmus amplitude (°)	−0.15	0.27	0.76	Horizontal foveal pit diameter	0.001	0.65	0.53
				Foveal shape factor	5.69	0.60	0.56
Waveform shape factor	−0.19	0.14	0.87	Horizontal foveal pit diameter	−0.0003	−0.16	0.88
				Foveal shape factor	−0.04	0.39	0.71
VA (logMAR)	−0.14	0.32	0.74	Horizontal foveal pit diameter	−0.0009	−0.53	0.61
				Foveal shape factor	−0.03	0.31	0.76

Each model met the assumptions of linear regression. Coefficients, *t*-statistics, and *P* values are presented for each predictor. Negative adjusted *R*² values indicate poor model fit.

## Discussion

The present study demonstrates that adults with horizontal IN have a foveal pit that has a significantly more horizontally elongated shape compared to controls (foveal shape factor 0.89 vs. 0.96; *p* = 0.006, BF_10_ = 4.14). We also confirm the existence of slight horizontal foveal elongation in normally-sighted individuals, in agreement with Tick et al.^9^

Our results suggest that early-onset nystagmus may have a direct impact on foveal development, because IN eye movements develop postnatally in tandem with the fovea. Alternatively, of course, the results could also suggest that horizontal elongation of the fovea arises first and independently of IN, which in turn affects the development of the predominantly horizontal IN eye movements. It is important to note that the direction of any causal relationship between these two elements cannot be definitively characterized by this study alone, in which we chose to image only the eye with better VA. It would however be interesting to examine foveal shape in amblyopic fellow eyes of people with IN: if horizontal relative elongation was *not* present in amblyopic fellow eyes, this would imply that foveal shape arises as an adaptation to nystagmus, rather than the nystagmus resulting from pre-existing foveal abnormality.

It had previously been assumed that motion blur resulting from nystagmus would be sufficient to reduce visual clarity,^41^^,^^42^ but more recent evidence^43^^,^^44^ indicates that motion blur does not limit VA in adults with IN (although this does not rule out the possibility that motion blur limits VA during infancy, leading to amblyopia). Considering this evidence of foveal elongation, we propose that the reduced VA in idiopathic IN could, at least in part, be explained by the normal number of retinal ganglion cells being spread over a wider area, thus limiting resolution. It is worth clarifying however that our results did not find a significant direct relationship between the degree of foveal elongation and VA. The interactions between the development of IN and foveal structure are likely complex, and additional work will be required to explore this relationship further. Foveal elongation might be reflected at the cellular level, with potential differences in photoreceptor density horizontally and vertically. It is also worth noting that although we use the term “horizontal elongation,” our findings could also be characterized as “vertical compression.” In the absence of a known mechanism that would cause elongation (or compression), the relative terms are analogous to one another, and causation is not implied.

RtFP was higher in participants with IN compared to controls, whereas foveal pit depth and foveal pit diameter (both horizontal and vertical) were lower in participants with IN compared to controls. These results agree with previous studies in which a shallower foveal pit depth^40^^,^^45^ and increased RtFP^45^ were also found in individuals with idiopathic IN without genetic mutations. These findings could indicate a mild structural foveal pathology in people with idiopathic IN.

Multiple regression models for horizontal IN amplitude, waveform shape factor, and VA (with horizontal foveal pit diameter and foveal shape factor as predicting variables) were not significant. As the IN waveform evolves throughout infancy, waveform parameters in adulthood do not necessarily represent those in infancy, while the fovea is developing. This could explain why the findings suggest a lack of a relationship between waveform parameters and foveal pit parameters. However, the lack of statistical significance in these models could also be due to the sample size. The most pertinent comparison of foveal shape factor yielded a Cohen's *d* of −1.01 (95% CI, −1.81 to −0.15)—a large point estimate by conventional benchmarks but with a wide confidence interval. These results should therefore be viewed as hypothesis-generating rather than conclusive.

A limitation of this study is the lack of complete genetic homogeneity within the idiopathic IN cohort. Although all participants included in the final analysis had a clinical diagnosis of idiopathic IN, confirmatory genetic testing was not completed in all cases. To improve diagnostic precision, genetic testing was offered, and individuals found to have pathogenic mutations associated with foveal structural abnormalities were excluded. This approach enhanced methodological rigor and allowed for the exclusion of at least some individuals who would have otherwise been wrongfully included. However, it is important to acknowledge that achieving full genetic homogeneity in idiopathic IN cohorts is inherently challenging, and future studies must carefully balance the need for genetic specificity with the practical demands of recruiting adequately sized and representative samples. A further limitation is that, although the long-wavelength (λc 1040 nm) SD-OCT system used here has been well characterized in previous retinal imaging studies, it has not previously been applied in a nystagmus cohort, and device-specific reproducibility or reliability data in this population are not yet available. However, the high imaging rate and single B-scan acquisition for both horizontal and vertical foveal images likely helped to reduce motion artefacts, as well as the additional analyses undertaken to account for potential effects of the nystagmus waveform on image measurements. Even so, we acknowledge that future work should include formal reproducibility testing of this system in participants with nystagmus.

Although this report is the first direct measure of horizontal/vertical foveal pit geometry in nystagmus, it should be noted that a previous study of albinos (who almost always have nystagmus) examined the thickness of ganglion cell layers across the horizontal and vertical meridians of the fovea. Their results also appear to suggest relative horizontal foveal elongation, although this was not directly measured or reported.^46^

The present study demonstrates that foveal pit diameter is significantly horizontally elongated in participants with horizontal IN as compared to age, sex, and ethnicity-matched controls. Because IN develops in tandem with the fovea, this suggests that IN and foveal development could impact on each other, providing a possible basis for reduced VA, particularly in individuals with idiopathic IN for whom no comorbid visual condition can explain poor vision. Future adaptive optics studies should investigate whether differences in foveal pit diameter are apparent at the level of photoreceptor density and determine the timeline of this “elongation” in an infant population. The results of this study have implications for understanding the relationship between eye movements and visual development and provide evidence for plasticity of the human fovea.
